# Elective “True Day Case” Laparoscopic Inguinal Hernia Repair in a District General Hospital: Lessons Learned from 1000 Consecutive Cases

**DOI:** 10.1155/2018/7123754

**Published:** 2018-06-03

**Authors:** A. Solodkyy, M. Feretis, A. Fedotovs, F. Di Franco, S. Gergely, A. M. Harris

**Affiliations:** Department of Upper Gastrointestinal Surgery, Hinchingbrooke Hospital, Hinchingbrooke Park, Huntingdon, Cambridgeshire PE29 6NT, UK

## Abstract

**Introduction:**

Laparoscopic inguinal hernia repair (LIHR) is ideal for day case surgery. It is recommended that at least 70% should be day cases as a measure of cost-effectiveness. The aims of this study were to (i) assess the rate of true day case (TDC) surgery and (ii) identify predictors associated with unexpected overnight stay (UOS).

**Methods:**

Data was collected prospectively on 1000 consecutive elective LIHR performed in a District General Hospital (DGH) over a 7-year period. Data was collected on baseline patient demographics, ASA grade, and intraoperative details. A multivariate analysis was performed in order to identify predictors of UOS.

**Results:**

1000 patients (927 males) underwent elective LIHR. Mean age was 57.3±15.2 years. 915 patients were planned as day case procedures. 822/915 day cases (89.8%) were discharged on the same day and 93 (10.2%) stayed overnight unexpectedly. Patient age, duration of procedure, and patient slot in the operating list were found to be independent predictors (p<0.05) of UOS.

**Conclusion:**

Our results demonstrate that LIHR is a “true” day case procedure in a DGH. Although some factors associated with UOS cannot be altered, careful patient selection and operating list planning are of paramount importance in order to minimise the burden on healthcare resources.

## 1. Introduction

In 2009, the European Hernia Society (EHS) published guidelines with indications for laparoscopic and open inguinal hernia repair. They recommend a laparoscopic approach to be considered for bilateral hernias and recurrent hernias after previous anterior repair and for all females. A Lichtenstein repair is recommended for large scrotal hernias, after previous abdominal surgery, and when general anaesthesia is not advised. Primary unilateral hernias can be repaired using a Lichtenstein or laparoscopic approach depending on surgeon expertise [1]. In the United Kingdom (UK) the National Institute for Clinical Excellence (NICE) has revised their guidance on the surgical management of inguinal hernias to recommend that laparoscopic repair be considered for first-line management of unilateral primary hernias [2]. The change in the recommendation resulted from the findings of a meta-analysis which highlighted the lower rate of wound-related complications, lower postoperative pain symptoms experienced by patients, and earlier return to daily activities [3]. Laparoscopic hernia repair has the advantage by reducing the time and effort that a patient's family or other carers devote to care, following discharge from hospital [4]. Stylopoulos et al. concluded the laparoscopic hernia repair is cost effective approach and associated with higher quality-of-life benefits at lower cost in their huge study population [5].

Despite the potential role LIHR has to play in the management of patients with symptomatic inguinal hernias, the procedure has not gained universal acceptance amongst surgeons. In the United States (US) only 14–19% of inguinal hernia operations were reported to be repaired using the laparoscopic approach [6, 7]. One population-based analysis in Florida revealed that even the majority of recurrent hernias was being repaired using an open approach [6]. In the UK the trend regarding the repair of choice for inguinal hernias appears to be similar to the US with only 4.9% of all hernia repairs being performed using the laparoscopic approach in Scotland [8]. We were unable to find published data on the UK-wide proportion of laparoscopic hernia repair (versus open) but anecdotally it is thought to be around 20-25%. The low uptake has been thought to be due to the fact that LIHR is a challenging procedure with a steep learning curve and the benefits for patients are still being debated. One concern surgeons have when considering a switch to routine laparoscopic repair of primary inguinal hernias is the increased operating time indicated in some of the trials. A meta-analysis of eight randomised controlled trials reported in the NICE guidelines quotes a mean increase in operating time of 7.9 minutes for TEP repair, when compared to standard open mesh repair [2]. Although this increase in operating time is not great, when the large number of repairs performed annually is considered (10 hernia repairs per 10,000 population per year in the UK), even a small increase could have a significant impact on health care resources, efficiency, and waiting lists in Day Surgery Units. It is however recognised that with experience the duration of the laparoscopic technique can reduce to parity with open surgery (personal data). Within the National Health Service (NHS), there is a drive to consider day surgery as first-line, rather than inpatient surgery, with the Department of Health recommendation that 75% of all elective surgeries be performed as day case procedures [9].

The aims of this report were (i) to assess the “true” rate of LIHR performed as “day cases”, (ii) to identify the immediate postoperative complications experienced by patients, and (iii) to identify predictors of unexpected patient overnight stay in a DGH.

## 2. Methods

Data was prospectively collected on 1000 consecutive patients who underwent LIHR using the trans-abdominal preperitoneal (TAPP) approach in our institution's Day Case Unit (DCU) over a 7-year period (2010-2017). Data was collected on baseline patient demographics, preoperative body mass index (BMI), American Society of Anaesthetists (ASA) patient grade, level of experience of operating surgeon, duration of procedure, laterality of the hernia, and immediate postoperative complications. Day case surgery was defined as ‘*admission and discharge on the same day as surgery, with day case surgery as the intended management'*, as recently described by Anderson et al. [10].

Continuous variables are reported as “mean” values and Standard Deviation (SD). Categorical variables are reported as absolute frequencies/percentages (%). Continuous variables were compared (univariate analysis) using the Mann–Whitney or independent samples t-test, depending on the distribution of the data. The chi-square or Fisher's exact test was used for comparison purposes between categorical variables. A binary, logistic regression model was developed in order to identify independent predictors of unexpected patient overnight stay. A p-value <0.05 was considered to be statistically significant. The Statistical Package for the Social Science (SPSS, version 24.0 Armonk, NY) was used for analysis purposes.

*Surgical Technique*

*3-Port Placement*. 1 x 10mm supraumbilical, 2 x 5mm left and right lateral. Patient placed in slight head-down position.

*Peritoneum*. Transverse peritoneal incision was extended medially to lateral umbilical ligament, 3-4cm above hernia deficit, using diathermy scissors; preperitoneal space was developed with blunt dissection. Hernia sac reduced using blunt dissection; vas and testicular vessels were clearly seen and preserved.

*Mesh*. 15 x 10cm Premilene™ mesh [*B. Braun: Melsungen, Germany*] was placed in preperitoneal space and fixed with Securestrap™ [*Ethicon: New Jersey, USA*] tacks placed at lower medial, upper medial, and upper lateral corners, avoiding lower lateral corner (so-called ‘triangle of sorrow'). Peritoneum was closed with tacks avoiding inferior epigastric artery.

*Closure*. ‘J' PDS to deep fascia, 3/0 prolene to skin.

## 3. Results

During the study period, 1000 patients (927 males) underwent elective LIHR in our institution's day case surgery unit. Mean age of the patients at the time of surgery was 57.3±15.2 years. Within this group 761 (76.1%) cases were unilateral and 239 (23.9%) cases were bilateral hernia repairs. The median body mass index (BMI) (kg/m^2^), as recorded on patients' visit to our institution's preassessment clinic prior to surgery, was 26.3 ±3.5. The patients' American Society of Anaesthetists (ASA) patient grade was as follows: ASA 1 n= 454 (45.4%); ASA 2 n= 487 (48.7%); and ASA 3 = 59 (5.9%), respectively. Mean operating time for performing the LIHR was 60.9 ± 21.3 minutes. This includes unilateral and bilateral cases. Only 5 out of 1000 (0. 5%) laparoscopic procedures were converted to open surgery due to technical difficulty (e.g., irreducibility of incarcerated hernia). Patient baseline and operative characteristics are summarised in Table 1.

Nine hundred and fifteen patients (91.5%) were scheduled to undergo LIHR as “day case admissions”, whereas 85 patients (8.5%) were prebooked for overnight hospital stay case by case taking into consideration multiple factors (patient premorbid status/social reasons). These patients were excluded from further analysis.

With regard to those patients (n=915) who were scheduled to be discharged on the day of their procedure, 822 out of 915 (89.8%) achieved the TDC target, whereas 93 out of 915 patients (10.2%) required UOS due to a variety of reasons, summarised in Table 2. Forty-four patients (47.3%) experienced urinary retention in the immediate postoperative period and a urethral catheter was inserted. One patient was catheterised for urinary retention but was discharged home on the same day with catheter. Anaesthetic issues such as prolonged postoperative nausea and vomiting, hypotension, and desaturation were documented in 23 patients (24.7%). The other common reasons for UOS were postoperative pain in 16 patients; 5 patients experienced immediate postoperative bleeding causing haematoma and were admitted for observation; 5 patients stayed due to unexpected other reasons.

### 3.1. TDC Versus UOS (Univariate Analysis)

Patients who required an unexpected overnight hospital stay were older (63.22±13.83 years) compared to the TDC group (55.5±14.8 years, p<0.0001). Furthermore, the mean operating time was significantly longer in the UOS group (66.8±24.2) compared to the TDC patient group (60.3±21.0 min, p=0.0091). The LIHR took more than 90 minutes in 15/93(16.1%) of patients in the UOS group, whereas only 78/822(9.4%) in the TDC group had procedure which was longer than this time. The difference, however, did not reach statistical significance (p=0.068).

The majority of patients [68/93(73.1%)] in the UOS group were operated in the second half of the operating list when compared to the TDC group 447/822 (54.4%, p=0.0009). This also explains why significant proportion of UOS patients left recovery after 18:30 hrs and did not have adequate time to recover prior to the same day discharge, when compared to those who were discharged on the same day [23/93 (24.7%) versus. 50/822(6%), p<0.0001].

There was no difference in the intraoperative complications experienced by patients of the two groups; however, immediate postoperative complications such as urinary retention were significantly higher in the UOS group (47.3% versus. 0.0%; p<0.0001). The primary surgeon was of training grade in 84 out of 822 cases (10.2%) in the TDC group versus 6 out of 93 procedures (6.5%) in the UOS group (p>0.05). The proportion of patients who underwent bilateral LIHR in the UOS group (28/93, 30%) was comparable to that of the TDC group [178/822, 21.6% (p=0.067)]. Finally, there was no significant difference between the two groups in terms of patient gender, BMI values, and ASA grade (p>0.05).

### 3.2. Independent Predictors of Unexpected Overnight Stay

We created a logistic regression model in order to identify independent predictors of unscheduled overnight patient stay following LIHR. The following variables were entered into the model: patient age, ASA grade, BMI, duration of procedure, hernia laterality (i.e., bilateral versus. unilateral), grade of operating surgeon, and patient slot in the operating list (i.e., a.m. versus p.m.). The following 3 variables were independent predictors of unexpected overnight patient stay: patient age, duration of procedure, and patient slot in the operating list. The univariate and multivariate analysis is shown in Table 3.

## 4. Discussion

In this report, we present data from 1000 consecutive patients undergoing elective LIHR in a DGH. In our experience, LIHR can be safely offered as a day case procedure thereby minimising the financial burden on healthcare resources. Furthermore, we identified three factors (patient age, duration of surgery, and patient slot in the operating list) as predictors of UOS. The authors suggest that taking these factors into account early in the preoperative stage could lead to maximal utilisation of operating list and would also lead to further reduction of healthcare expenses.

Groin hernia repairs are amongst the most commonly performed general surgical operations with over 71,000 inguinal and femoral hernias repairs carried out in England in 2014/15. Previous economic analyses have estimated that, in England alone, surgical repair of inguinal hernias utilised over 100,000 NHS bed-days of hospital resources. The British Association of Day Surgery has suggested that 80% of inguinal hernia repairs should be carried out as day case procedures, regardless of the technique used. In 2014/15, 77.8% of primary inguinal hernia repairs (unilateral) were carried out as a day case, and rates varied from 67% to 88% across institutions. Furthermore, surgeons have a variety of approaches and materials in their armamentarium [2, 11]. It has long been established that LIHR can be performed safely and effectively as a day case procedure. However, the uptake of the technique has not been endorsed by many surgeons for a variety of reasons. A recent survey amongst members of the American Hernia Society revealed that just over 50% of surgeons utilise the laparoscopic approach for repair of inguinal hernias, with lack of training, increased operating time, and costs being the commonest reasons to opt for the open approach [12]. In our unit, the laparoscopic approach has been the method of choice for repairing symptomatic unilateral or bilateral inguinal hernias for the past 15 years. A typical straightforward unilateral hernia repair will take around 30-45 mins and bilateral 45-60 mins. As our results demonstrate, operation time achieves equivalence (to open surgery) with experience; conversion rates to open surgery have been extremely low and morbidity associated with the procedure has been minimal.

The criteria for selecting patients suitable for day case surgery have changed over the past 15 years as a result of the pressure on healthcare resources, lack of hospital capacity, and the obesity pandemic. However, the current practice for many units in the United Kingdom (UK) is to routinely book patients with high BMI (>30kg/m^2^), diabetic patients, and all those classified ASA 3 for an overnight hospital stay. NHS Modernisation in 2002 raised the BMI limit for day surgery procedures, from 30kg/m^2^ in 1992 (Royal College of Surgeons recommendation) to 35 kg/m^2^; however, other professional bodies (Association of Anaesthetists of Great Britain and Irelands) recommend that the patients are not excluded from day surgery on BMI alone [10]. Our experience over a seven-year period was that BMI alone should not be a limiting factor for the surgeon to perform LIHR as day case procedure. Nevertheless, our standard practice as a unit is to encourage obese patients to lose weight prior to elective surgery.

According to British Hernia Society (BHS), the strict patient selection criteria are becoming less common and, in principle, an inguinal hernia repair as day surgery could be potentially offered for virtually every patient who has satisfactory care at home [13]. As our results demonstrate, ASA grade should not be a limiting factor for offering patients LHR as day case, provided that patients attend a preassessment clinic. In a large American cohort study, the costs of an inguinal hernia repair in a clinical setting were found to be 56% higher than those for day surgery [14]. Also, in Germany, day case surgery has been reported to generate lower costs [15]. It is therefore evident, in an era where healthcare resources are not unlimited, that accurate preoperative assessment is essential in order to keep expenses as low as possible.

Similar to others, our departmental policy is to repair bilateral inguinal hernias laparoscopically from a cost-utility and patient perspective [16–20]. Our analysis did not reveal hernia laterality (i.e., bilateral versus unilateral repair) to be an independent predictor of UOS. However, the regression model identified duration of procedure as an independent predictor of UOS. Changes in patient physiological parameters whilst under anaesthesia along with difficult dissection at the level inguinal region could be potential explanations for this finding. Furthermore, given that laparoscopic hernia repair has a relatively slow learning curve, as previous reports have demonstrated, one might expect a difference in the rates of UOS between qualified surgeons and surgeons in training. Comparison of the figures did not show a statistically significant difference. This can be explained by that fact that as a laparoscopic training unit the consultants are present for all training hernia cases and will step in if difficulties are encountered, which helps to control operation time and minimises morbidity/complications. A few trainees become very proficient at this procedure requiring less intervention (as would be expected) and as such their cases will not unduly affect the UOS rate.

In our day case surgery unit we apply only one absolute exclusion criterion which is the absence of a responsible adult to stay with the patient for the first 24 hours following discharge. We suggest that criteria other than social should be considered relative and selection should be done on a case by case basis. However, patients older than 60 years or/and have other significant comorbidities (e.g., diabetes) should be listed for morning rather than afternoon procedure to allow for longer recovery time before discharge. Both these variables were independent predictors of UOS in our patient cohort and highlight the necessity of preoperative patient planning in order to minimise costs and the impact on bed availability.

## 5. Conclusion

Our results demonstrate that LIHR can be offered as a “true” day case procedure with high same day discharge rate of around 90% in a DGH. Although some factors associated with UOS cannot be altered, careful patient selection and operating list prioritisation are of paramount importance in order to minimise number of unexpected overnight stay. We recommend that patients who are older and have multiple comorbidities should be booked on a morning list to allow adequate recovery time and thus increase the chance of same day discharge. Patients with BMI ≥ 35 or ASA 3 who are routinely scheduled for overnight stay in many units are in our experience often suitable for day case surgery with careful planning, good preoperative advice, and established communication channels, should they require advice or support after discharge. These measures may increase the overall rate of true day case surgery for LIHR and have a significant cost benefit by reducing the added bed pressures and financial costs of overnight or further hospital stay.

## Figures and Tables

**Table 1:** Baseline characteristics of the study's population.

	**Total (N=1000)**
Age*∗* (years)	57.3±15.2

Gender† (Male: Female)	927:73

Single side hernia†	761(76.1%)

Bilateral hernia†	239(23.9%)

BMI*∗* (Kg/m^2^)	26.3±3.5

ASA grade†	
(i) 1	454(45.4%)
(ii) 2	487(48.7%)
(iii) 3	59 (5.9%)

*∗*→ values as mean/SD and †→ values as frequencies/ percentages.

**Table 2:** Reasons for unexpected overnight hospital stay (n=93).

**Reason**	**Number of Patients (**%**)**
Retention of urine	44 (47.3%)

Complications related to general anaesthesia/ slow recovery	23 (24.7%)

Post-operative pain	16 (17.2%)

Bleeding post operatively	5 (5.4%)

Other	5 (5.4%)

**Table 3:** Comparison (univariate and multivariate analysis) of baseline demographic and operative characteristics of “true day cases” (TCD) and patients who required an unexpected overnight stay (UOS).

**Parameter **	**TDC (N=822)**	**UOS (n=93)**	**Univariate Analysis**	**Multivariate Analysis**
Age years *∗*(Mean ± SD)	55.5±14.8	63.2±13.83	p<0.0001	**P=0.0001**

Age *∗*≥60	377(45.9%)	63(67.8%)	p<0.0001	

Male gender †	769(93.5%)	84 (90.3%)	p=0.2723	

BMI*∗* (Kg/m2)	26.4±3.5	25.8±3.5	p>0.05	P=0.110

BMI*∗* ≥ 35 Kg/m^2^	18(2.2%)	2 (2.1%)	p=0.3457	

ASA1/2 vs ASA3	798/23	87/6	p=0.1126	P=0.582

Mean operation time (mins)*∗*	60.3±21.0	66.8±24.2	p=0.0091	**p=0.021**

Length of procedure (>90 minutes†)	78 (9.4%)	15 (16.1%)	p=0.068	

Time leaving recovery (after 18:30 hr) †	50(6%)	23 (24.7%)	p<0.0001	

Recovery time (min) *∗*	68.7±31.4	80.3±46.9	p=0.0226	

Timing of procedure (PM list) †	447(54.4%)	68 (73.1%)	p=0.0009	**p=0. 001**

Immediate post-operative complications (urine retention, bleeding) †	1 (0.00%)	49(52.7%)	p<0.0001	

Operations by trainee(SAS/Registrar) †	84 (10.2%)	6(6.5%)	p>0.05	P=0.375

Bilateral/unilateral	178/644	28/65	p=0.067	p=0.267

*∗*→ values as mean/SD and †→values as frequencies/ percentages.

## Data Availability

The data used to support the findings of this study are available from the corresponding author upon request.
